# Geriatric nutritional risk index as a prognostic marker of pTNM-stage I and II esophageal squamous cell carcinoma after curative resection

**DOI:** 10.18632/oncotarget.27670

**Published:** 2020-07-21

**Authors:** Noriyuki Hirahara, Takeshi Matsubara, Yusuke Fujii, Shunsuke Kaji, Ryoji Hyakudomi, Tetsu Yamamoto, Yuki Uchida, Yoshiko Miyazaki, Kazunari Ishitobi, Yasunari Kawabata, Yoshitsugu Tajima

**Affiliations:** ^1^Department of Digestive and General Surgery, Shimane University Faculty of Medicine, Izumo, Shimane 693-8501, Japan

**Keywords:** geriatric nutritional risk index, esophageal squamous cell carcinoma, curative esophagectomy, cancer-specific survival, pathological tumor-node-metastasis

## Abstract

The geriatric nutritional risk index (GNRI) is associated with mortality in several malignancies. We retrospectively analyzed whether the GNRI can predict long-term outcomes in 191 patients with esophageal squamous cell carcinoma (ESCC) after curative esophagectomies by evaluating their cancer-specific survival (CSS). In multivariate analyses, serum albumin (hazard ratio [HR], 2.498; *p* = 0.0043), GNRI (HR, 1.941; *p* = 0.0181), pathological tumor-node-metastasis (pTNM) stage (HR, 3.884; *p* < 0.0001), and tumor differentiation (HR, 2.307; *p* = 0.0066) were independent prognostic factors for CSS.

In pTNM stage I, multivariate analysis identified C-reactive protein (HR, 7.172; *p* = 0.0483) and GNRI (HR, 5.579; *p* = 0.0291) as independent prognostic factors for CSS. In univariate analyses in pTNM stages II and III, only low GNRI (*p* = 0.0095) and low serum albumin levels (*p* = 0.0119), respectively, were significantly associated with worse CSS. In patients with low GNRI, CSS was significantly worse than in those with normal GNRI (*p* = 0.0011), especially in pTNM stages I (*p* = 0.0044) and II (*p* = 0.0036) groups, but not in stage III group (*p* = 0.5099).

Preoperative GNRI may sort patients into low- or high-risk groups for shorter CSS, especially in those with pTNM stage I and II ESCC.

## INTRODUCTION

Patients with advanced esophageal cancer presenting with dysphagia often experience malnutrition as well as impairment of performance status and quality of life. Disease-related malnutrition may suppress anti-tumor immunity and is an independent predictor of worse clinical outcomes [1]. The geriatric nutritional risk index (GNRI) was established by Bouillanne *et al*. as a risk screening tool for nutrition-related morbidity and mortality [2]. The GNRI is a widely used, simple, and objective measure, calculated using the body mass index (BMI) and serum albumin levels. Serum albumin is a clinically relevant indicator of nutritional status, such as malnutrition and cachexia [3, 4]. Additionally, hypoalbuminemia is often associated with systemic inflammation and impaired host immune responses [5, 6]. Systemic inflammation promotes tumor progression and metastasis [7]. Concurrently, the BMI, which is calculated using body weight and height, is also used to assess the nutritional status of an individual. A low BMI is known to be a negative prognostic factor in several cancers [8, 9].

Recently, it has been widely accepted that the GNRI was strongly associated with mortality in elderly hospitalized patients and in patients with various cancers [10–12]. However, to the best of our knowledge, there have been few reports on the prognostic significance of the GNRI in patients with esophageal squamous cell carcinoma (ESCC). In this study, we have investigated whether the GNRI is a useful predictor of long-term survivals in patients with ESCC who underwent a curative esophagectomy.

## RESULTS

### Association between the GNRI and various clinicopathological features

The association between the GNRI and clinicopathological features in all patients in this study is shown in Table 1. Based on the GNRI cutoff value of 97.1, 81 (42.4%), and 110 (57.6%) patients were classified as having low and normal GNRIs, respectively. There was a significant association between the GNRI and various clinicopathological factors, such as the BMI (*p* < 0.0001), tumor size (*p* < 0.0077), depth of tumor (*p* < 0.0001), the pathological tumor-node-metastasis (pTNM) stage (*p* = 0.0038), serum squamous cell carcinoma (SCC) antigen (*p* = 0.0001), serum albumin levels (*p* < 0.0001), and C-reactive protein (CRP) levels (*p* < 0.0001).

**Table 1:** Relationships between GNRI and clinicopathological features in all patients with ESCC

Characteristics	Total patients	GNRI		
		< 97.1 (*n* = 81)	≥ 97.1 (*n* = 110)	*p* value
Age (years)		67 (48–84)	66 (47–85)	0.3170
Sex				0.1267
Male	169	75	94	
Female	22	6	16	
BMI		18.5 (13.1–26.3)	22.2 (16.5–31.8)	< 0.0001
WBC		5730 (2560–12240)	5435 (2710–17140)	0.7366
Neutrophil		3425 (849–9420)	3257 (860–15169)	0.5700
Lymphocyte		1410 (160–3470)	1600 (380–3340)	0.0795
Platelet		24.4 (8.7–49.5)	21.6 (11.2–41.8)	0.0574
Location of tumor				0.6629
Ce	13	7	6	
Ut	10	4	6	
Mt	79	36	43	
Lt	69	28	41	
Ae	20	6	14	
Tumor size (mm)		5.0 (0.2–70.0)	3.7 (0.5-48.0)	0.0077
Depth of tumor				< 0.0001
T1a–1b	81	23	58	
2	16	4	12	
3	75	39	36	
4a–4b	19	15	4	
Lymph node metastasis				0.0901
N0	102	40	62	
N1	53	28	25	
N2	21	5	16	
N3	15	8	7	
Pathological TNM stage				0.0038
Ia–Ib	73	20	53	
IIa–IIb	41	20	21	
IIIa–IIIc	77	41	36	
Operation time (min)		647 (156–1073)	653 (196–1258)	0.8831
Intraoperative blood loss (ml)		630 (0–10000)	375 (0–3000)	0.0842
SCC antigen		1.3 (0.1–20.8)	0.8 (0.1–6.0)	0.0001
Albumin		3.6 (2.3–4.6)	4.2 (3.3–4.9)	< 0.0001
CRP		0.20 (0.01–6.02)	0.20 (0.01–4.00)	< 0.0001

### Cox regression analysis of cancer-specific survival in all patients

Univariate analyses identified that low serum albumin levels (*p* = 0.0005), high CRP (*p* = 0.0021), low GNRI (*p* < 0.0001), large tumor size (*p* = 0.0026), advanced pTNM stage (*p* < 0.0001), and not being well differentiated (*p* = 0.0276) were significantly associated with worse cancer-specific survivals (CSS). Multivariate analysis revealed that the serum albumin levels (hazard ratio [HR], 2.498; 95% confidence interval [CI], 1.333–4.684; *p* = 0.0043), GNRI (HR, 1.941; 95% CI, 1.120–3.365; *p* = 0.0181), pTNM stage (HR, 3.884; 95% CI, 2.229–6.769; *p* < 0.0001), and tumor differentiation (HR, 2.307; 95% CI, 1.262–4.220; *p* = 0.0066) were the independent prognostic factors for CSS in all patients (Table 2).

**Table 2:** Prognostic factors for cancer-specific survival in all patients with ESCC

Variables	Patients (*n* = 191)	Category or characteristics	Univariate analyses			Multivariate analyses		
			HR	95% CI	*p* value	HR	95% CI	*p* value
Gender	22/169	(female/male)	1.310	0.599–2.867	0.4993			
Age	81/110	(<65 / ≥65)	0.961	0.594–1.554	0.8713			
BMI	144/47	(≥18.5 / <18.5)	1.616	0.946–2.758	0.0787			
Alb	159/32	(≥3.5 / <3.5)	2.747	1.557–4.845	0.0005	2.498	1.333–4.684	0.0043
CRP	148/43	(<0.5 / ≥0.5)	2.262	1.344–3.807	0.0021	1.205	0.673–2.157	0.5300
GNRI	110/81	(≥97.1 / <97.1)	2.879	1.764–4.698	< 0.0001	1.941	1.120–3.365	0.0181
Tumor size	54/136	(<3 / ≥3)	2.704	1.415–5.170	0.0026	1.229	0.614–2.461	0.5600
pTNM stage	114/77	(I, II / III)	4.600	2.747–7.704	< 0.0001	3.884	2.229–6.769	< 0.0001
Differentiation	64/127	(well / other)	1.908	1.074–3.390	0.0276	2.307	1.262–4.220	0.0066
SCC	139/52	(<1.5 / ≥1.5)	1.546	0.921–2.595	0.0990			

### The CSS and GNRI in all patients

**Figure 1:**
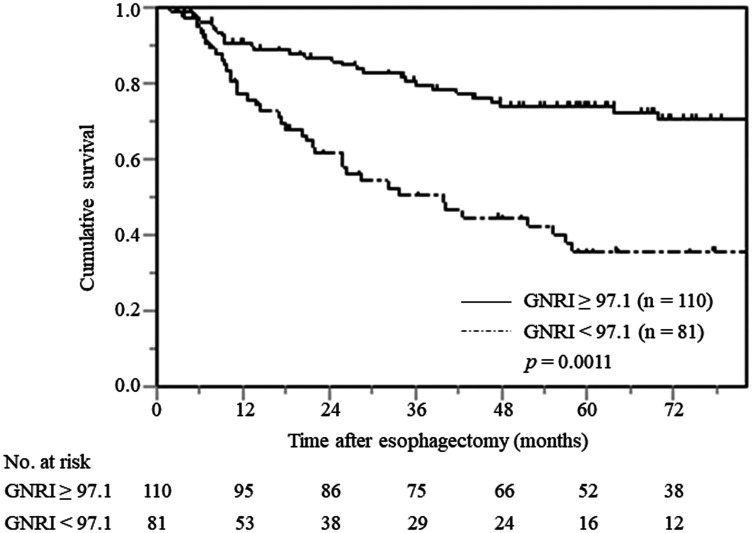
Cancer-specific survival curves in all patients with ESCC stratified by preoperative GNRI.

In patients with low and normal GNRIs, the 3-year CSS rates were 51.3% and 79.3%, respectively, and the 5-year CSS rates were 36.9% and 73.8%, respectively. The log-rank test demonstrated that patients with low GNRIs had significantly worse prognoses, with respect to their CSS, than those with normal GNRIs (*p* = 0.0011) (Figure 1). Similarly, patients with low GNRIs had a significantly worse prognosis in terms of overall survival (OS) than those with normal GNRIs (*p* < 0.001) (Supplementary Figure 1).

### The GNRI value relative to pTNM stage

**Figure 2:**
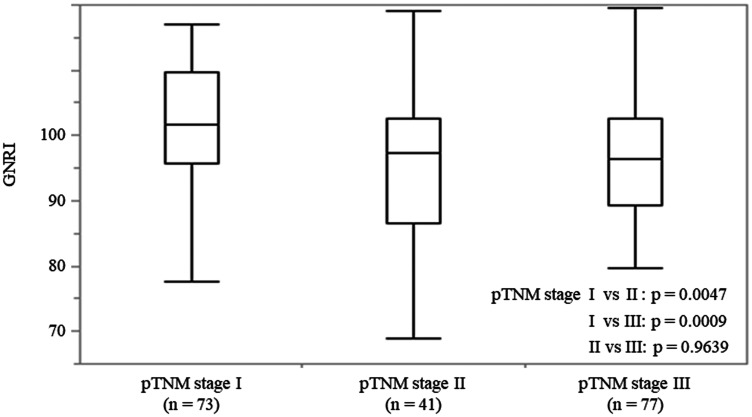
The GNRI values in patients with ESCC at each pTNM stage. On each box, the central mark indicates the median, and the bottom and top edges of the box indicate the 25th and 75th percentiles, respectively. Capped bars indicate the minimum and maximum values, respectively.

The association between the GNRI and pTNM stage is shown in Figure 2. The mean preoperative GNRIs were 101.6 (range 69.8–117.0), 97.3 (range 68.8–119.1), and 96.4 (range 65.9–119.5) in patients with pTNM stage I, II, and III, respectively. There was a significant association between GNRI and pTNM stage in these patients (*p* = 0.0012).

### Association between the GNRI and clinicopathological factors in subgroups with pTNM stage I, II, and III

Based on the GNRI cutoff value of 97.1, 20 (27.4%) and 53 (72.6%) patients were classified as having low and normal GNRIs, respectively. There was a significant association between the GNRI and clinicopathological factors, such as the BMI (*p* < 0.0001), SCC antigen (*p* = 0.0019), and serum albumin levels (*p* < 0.0001) in patients with pTNM stage I (Table 3A).

**Table 3A:** Relationships between GNRI and clinicopathological features in patients with ESCC stage I

Characteristics	Total patients	GNRI		
		< 97.1 (*n* = 20)	≥ 97.1 (*n* = 53)	*p* value
Age (years)		70 (53–80)	66 (47–85)	0.1873
Sex				0.9920
Male	62	17	45	
Female	11	3	8	
BMI		18.1 (13.3 – 20.8)	22.3 (16.5–31.8)	< 0.0001
WBC		5595 (3350–10800)	5060 (2710–9940)	0.6922
Neutrophil		3383 (1100–9410)	2920 (860–7296)	0.4924
Lymphocyte		1470 (560–3470)	1593 (818–2600)	0.2846
Platelet		23.4 (14.2 – 35.6)	20.7 (11.2 – 30.9)	0.0987
Location of tumor				0.4399
Ce	3	1	2	
Ut	1	0	1	
Mt	32	12	20	
Lt	28	5	23	
Ae	9	2	7	
Tumor size (mm)		3.9 (0.2–7.0)	3.7 (0.5–48.0)	0.4574
Depth of tumor				0.8374
T1a–1b	61	17	44	
2	12	3	9	
3	0	0	0	
4a-4b	0	0	0	
Lymph node metastasis				
N0	73	20	53	
N1	0	0	0	
N2	0	0	0	
N3	0	0	0	
Operation time (min)		624 (156–891)	645 (344–1217)	0.5527
Intraoperative blood loss (ml)		415 (50–1840)	470 (0–3000)	0.6922
SCC antigen		1.1 (0.3–4.2)	0.6 (0.1–2.3)	0.0019
Albumin		3.7 (3.0–4.6)	4.2 (3.6–4.9)	< 0.0001
CRP		0.20 (0.02–1.82)	0.20 (0.01–3.26)	0.9231

In patients with pTNM stage II, 20 (48.8%) and 21 (51.2%) patients were classified as having low and normal GNRIs, respectively. There was a significant association between the GNRI and various clinicopathological factors, such as the BMI (*p* = 0.0123), white blood cell (WBC) (*p* = 0.0489), neutrophil count (*p* = 0.0446), serum albumin levels (*p* < 0.0001), and CRP (*p* = 0.0096) (Table 3B).

**Table 3B:** Relationships between GNRI and clinicopathological features in patients with ESCC stage II

Characteristics	Total patients	GNRI		
		< 97.1 (*n* = 20)	≥ 97.1 (*n* = 21)	*p* value
Age (years)		72 (55–84)	65 (50–77)	0.0732
Sex				0.6740
Male	36	18	18	
Female	5	2	3	
BMI		19.1 (13.1–24.4)	20.7 (17.9–27.5)	0.0123
WBC		4460 (3070–11360)	6120 (3920–11340)	0.0489
Neutrophil		2814 (1220–7168)	3672 (2347–8222)	0.0446
Lymphocyte		1144 (160–3400)	1600 (778–3330)	0.1337
Platelet		19.5 (9.4–39.7)	21.7 (13.6–41.8)	0.4416
Location of tumor				0.6296
Ce	3	1	2	
Ut	6	3	3	
Mt	16	9	7	
Lt	11	6	5	
Ae	5	1	4	
Tumor size (mm)		4.0 (2.0–8.0)	4.8 (0.5–38.0)	0.9792
Depth of tumor				0.2220
T1a–1b	17	6	11	
2	3	1	2	
3	21	13	8	
4a–4b	0	0	0	
Lymph node metastasis				0.1426
N0	21	13	8	
N1	19	7	12	
N2	1	0	1	
N3	0	0	0	
Operation time (min)		614 (270–1073)	633 (395–1258)	0.7741
Intraoperative blood loss (ml)		710 (0–2250)	560 (0–2620)	0.5228
SCC antigen		1.1 (0.1–6.2)	0.9 (0.1–2.1)	0.3018
Albumin		3.4 (2.8–4.2)	4.2 (3.6–4.9)	< 0.0001
CRP		0.33 (0.01–2.25)	0.14 (0.02–1.50)	0.0096

In patients with pTNM stage III, 41 (53.2%) and 36 (46.8%) patients were classified as having low and normal GNRIs, respectively. GNRI was significantly associated with BMI (*p* < 0.0001), tumor size (*p* = 0.0473), depth of tumor (*p* = 0.0071), lymph node metastasis (*p* = 0.0076), intraoperative blood loss (*p* = 0.0191), serum albumin levels (*p* < 0.0001), and CRP levels (*p* = 0.0019) (Table 3C).

**Table 3C:** Relationships between GNRI and clinicopathological features in patients with ESCC stage III

Characteristics	Total patients	GNRI		
		< 97.1 (*n* = 41)	≥ 97.1 (*n* = 36)	*p* value
Age (years)		64 (48–84)	67 (49–84)	0.5810
Sex				0.0534
Male	71	40	31	
Female	6	1	5	
BMI		18.5 (15.2–26.3)	22.3 (16.9–31.8)	< 0.0001
WBC		6700 (2560–12240)	5560 (3280–17140)	0.3025
Neutrophil		4270 (849–9420)	3408 (2268–15169)	0.3171
Lymphocyte		1480 (179–3390)	1605 (380–3340)	0.7170
Platelet		25.5 (8.7–49.5)	22.5 (14.5 – 38.0)	0.1269
Location of tumor				0.7522
Ce	7	5	2	
Ut	3	1	2	
Mt	31	15	16	
Lt	30	17	13	
Ae	6	3	3	
Tumor size (mm)		5.5 (2.0–70.0)	4.7 (1.3–32.0)	0.0473
Depth of tumor				0.0071
T1a–1b	3	0	3	
2	1	0	1	
3	54	26	28	
4a–4b	19	15	4	
Lymph node metastasis				0.0076
N0	8	7	1	
N1	34	21	13	
N2	20	5	15	
N3	15	8	7	
Operation time (min)		665 (230–986)	664 (196–1113)	0.9756
Intraoperative blood loss (ml)		660 (0–10000)	280 (0–1650)	0.0191
SCC antigen		1.3 (0.4–20.8)	1.1 (0.1–6.0)	0.3091
Albumin		3.6 (2.3–4.2)	4.2 (3.3–4.8)	< 0.0001
CRP		0.45 (0.01–6.02)	0.18 (0.02–4.00)	0.0019

### Cox regression analysis of CSS depending on pTNM stage

In patients with pTNM stage I, univariate analyses identified that low serum albumin levels (*p* = 0.0420), high CRP (*p* = 0.0153), and low GNRI (*p* = 0.0111) were significantly associated with worse CSS. Multivariate analysis revealed that CRP (HR, 7.172; 95% CI, 1.014–50.705; *p* = 0.0483) and GNRI (HR, 5.579; 95% CI, 1.191–26.133; *p* = 0.0291) were independent prognostic factors for CSS. In patients with pTNM stage II, univariate analyses confirmed that only low GNRI (*p* = 0.0095) was significantly associated with worse CSS; whereas, in patients with pTNM stage III, only low serum albumin levels (*p* = 0.0119) were significantly associated with worse CSS in the univariate analyses (Table 4).

**Table 4:** Prognostic factors for cancer-specific survival of patients with ESCC in each pTNM stage

Variables	Patients	Category or characteristics	Univariate analyses			Multivariate analyses		
			HR	95%CI	*p* value	HR	95%CI	*p* value
pTNM stage I	*n* = 73							
Gender	11/62	(female / male)	1.450	0.181–11.610	0.7264			
Age	23/46	(< 65 / ≥ 65)	1.358	0.340–5.434	0.6649			
BMI	60/13	(≥ 18.5 / < 18.5)	2.224	0.460–10.760	0.3205			
Alb	66/7	(≥ 3.5 / < 3.5)	5.148	1.061–24.965	0.0420	1.267	0.148–10.854	0.8290
CRP	67/6	(< 0.5 / ≥ 0.5)	7.636	1.477–39.482	0.0153	7.172	1.014–50.705	0.0483
GNRI	53/20	(≥ 97.1 / < 97.1)	5.537	1.477–20.759	0.0111	5.579	1.191–26.133	0.0291
Tumor size	54/139	(< 3 / ≥ 3)	0.920	0.247–3.428	0.9010			
Differentiation	28/45	(well/other)	4.455	0.557–35.629	0.1590			
SCC	60/13	(< 1.5 / ≥ 1.5)	2.436	0.504–11.764	0.2679			
pTNM stage II	*n* = 41							
Gender	5/36	(female / male)	0.723	0.160–3.275	0.6742			
Age	18/23	(< 65 / ≥ 65)	1.131	0.378–3.383	0.8261			
BMI	30/11	(≥ 18.5 / < 18.5)	1.399	0.427–4.577	0.5790			
Alb	30/11	(≥ 3.5 / < 3.5)	2.376	0.767–7.360	0.1335			
CRP	29/12	(< 0.5 / ≥ 0.5)	1.134	0.348–3.693	0.8344			
GNRI	21/20	(≥ 97.1 / < 97.1)	5.599	1.525–20.566	0.0095			
Tumor size	54/139	(< 3 / ≥ 3)	2.161	0.477–9.796	0.3179			
Differentiation	15/26	(well / other)	2.662	0.731–9.691	0.1374			
SCC	32/9	(< 1.5 / ≥ 1.5)	0.814	0.180–3.678	0.7889			
pTNM stage III	*n* = 77							
Gender	6/71	(female/male)	1.214	0.433–3.402	0.7122			
Age	36/41	(< 65 / ≥ 65)	1.101	0.612–1.978	0.7483			
BMI	54/23	(≥ 18.5 / < 18.5)	1.096	0.573–2.094	0.7823			
Alb	63/14	(≥ 3.5 / < 3.5)	2.640	1.239–5.626	0.0119			
CRP	52/25	(< 0.5 / ≥ 0.5)	1.242	0.665–2.321	0.4963			
GNRI	36/41	(≥ 97.1 / < 97.1)	1.218	0.677–2.193	0.5109			
Tumor size	54/139	(< 3 / ≥ 3)	1.431	0.512–4.000	0.4950			
Differentiation	21/56	(well / other)	1.252	0.632–2.478	0.5191			
SCC	47/30	(< 1.5 / ≥ 1.5)	0.806	0.441–1.473	0.4830			

### Relationship between the CSS and GNRI stratified by pTNM stage

**Figure 3:**
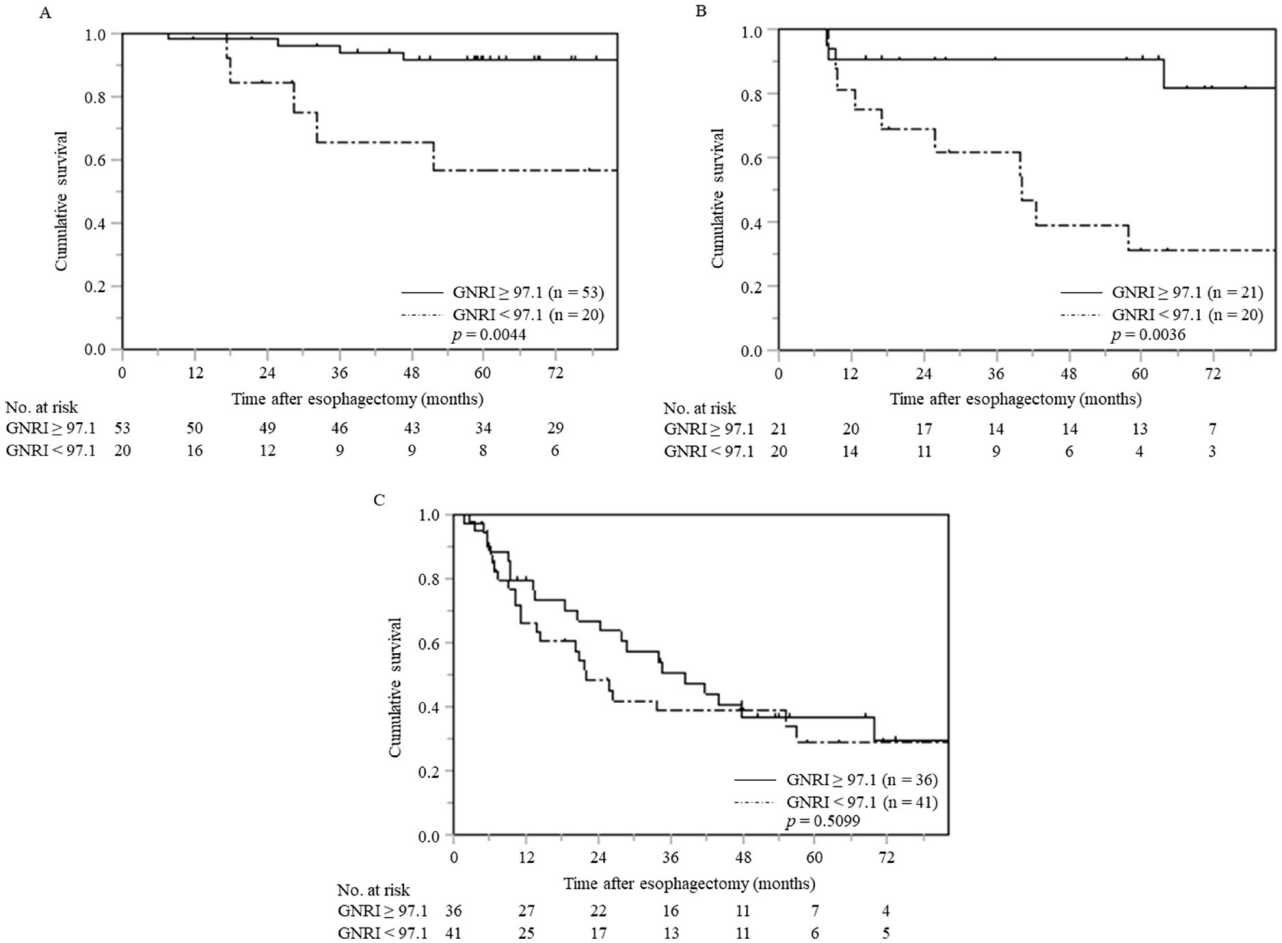
Cancer-specific survival curves in patients with ESCC at each pTNM stage stratified by preoperative GNRI (**A**) pTNM stage I (*n* = 73), (**B**) pTNM stage II (*n* = 41), (**C**) pTNM stage III (*n* = 77). Abbreviations: ESCC, esophageal squamous cell carcinoma; pTNM, pathological tumor-node-metastasis; GNRI, geriatric nutritional risk index.

The relationship between the CSS and GNRI in each patient group stratified by pTNM stage is shown in Figure 3. The 5-year CSS were significantly worse in patients with low GNRI than in those with normal GNRI, in both pTNM stage I (60.0% vs. 91.7%, *p* = 0.0044) and stage II (30.9% vs. 90.5%, *p* = 0.0036) groups. However, there was no significant difference in the 5-year CSS between patients with low and normal GNRIs (29.0% vs. 36.9%, *p* = 0.5099), respectively, in the pTNM stage III group. Similarly, patients with low GNRIs had a significantly worse OS than those with normal GNRIs (*p* < 0.001) in pTNM stage I (*p* < 0.0001) and stage II (*p* = 0.0020). In contrast, there was no significant association between GNRI and OS in patients with pTNM stage III (*p* = 0.1292) (Supplementary Figure 2A–2C).

## DISCUSSION

Many studies have identified that the tumor- or nutrition-associated factors and host immunity strongly affect the prognoses in patients with cancer [13–15]. Thus, this study aimed to clarify the significance of the GNRI for predicting long-term postoperative outcomes in patients with ESCC. Survival analysis in 191 patients with ESCC who received a curative esophagectomy revealed that patients with low GNRIs had significantly worse CSS than those with normal preoperative GNRIs. Similarly, there were significant differences in OS (Supplementary Figure 1). Additionally, multivariate analysis identified GNRI as an independent risk factor for CSS. The results obtained in this study are consistent with those shown in previous reports related to lung and gastric cancers [10–12].

The TNM staging is useful for predicting the survival in patients with different stages of cancer, but a precise prediction is often difficult among those with the same stage of cancer. Therefore, additional indicators are needed to distinguish different prognoses in the same TNM stage [16]. In this study, we focused on stage-stratified survival analysis with special attention to the GNRI in patients with ESCC. Multivariate analysis identified the GNRI as an independent predictor of CSS in patients with pTNM stage I ESCC. In patients with pTNM stage II ESCC, when compared to the normal GNRI, a low GNRI was associated with a significantly worse CSS in univariate analysis; no significant differences were noted among patients with pTNM stage III ESCC. These findings suggested that malnutrition could lead to worse CSS, even in patients with pTNM stage I and II ESCC after undergoing curative esophagectomy. The basic mechanism underlying the association between low GNRI and worse prognosis is unclear. From a nutritional perspective, patients with cancer tend to be malnourished and usually show a diminished anabolic response to nutritional repletion. Additionally, albumin synthesis may be suppressed even in patients with early stage cancers [17]. However, tumor-related factors may have a stronger impact than nutrition-related ones, on the prognoses in patients with advanced stages of cancer [18, 19]. The above-mentioned facts may explain why GNRI had no significant association with CSS in patients with pTNM stage III ESCC in our study. With tumor progression, tumor-related factors, such as invasion and migration, may gradually play a more prominent role in patient survival, when compared to nutrition-related factors [20–22].

Yamana *et al*. were the first to demonstrate that GNRIs can be a reliable predictor of OS in esophageal cancer patients and to report the clinical significance of GNRIs by preoperative treatment modality [23]. Subsequently, Migita *et al*. suggested that low preoperative GNRI was associated with a higher risk of cancer death in patients who underwent curative esophagectomy for esophageal cancer [24]. Wang also reported that GNRI was a predictive marker of OS and progression-free survival (PFS) in patients aged 70 years and older who have received radiation therapy or definitive concurrent chemoradiotherapy [25]. To the best of our knowledge, our study is the first report demonstrating that preoperative GNRI is an independent prognostic factor in patients with pTNM stage I ESCC and a significant prognostic marker in patients with pTNM stage II. Our results suggested that preoperative GNRI could be a useful marker to identify patients at high-risk for ESCC and that patients with pTNM stage I and II ESCC who have low GNRIs may require more careful follow-up even after undergoing curative esophagectomy.

Our study shows some limitations in confirming our findings. First, the present study cannot unambiguously determine the prognostic role of GNRI in ESCC patients because of its retrospective nature and small sample size. Second, nutritional and inflammatory assessments by parameters other than the GNRI were not conducted. Third, the GNRI was used as the only nutritional screening tool, and its utility was not compared with those of other common assessment tools, such as the assessment of prealbumin, sarcopenia, or the Glasgow prognostic scores (GPS). To overcome these limitations, further studies with a prospective nature and a more comprehensive design are warranted.

The survival outcomes in patients with ESCC after undergoing curative resection, even in those with earlier stages of ESCC, are insufficient when compared to those in patients with other malignancies [26]. The benefit of neoadjuvant and postoperative adjuvant chemotherapies for patients with pathological stage II or III ESCC has been established in Japan; the long-term outcomes are still unsatisfactory [27, 28]. More effective personalized treatment strategies for patients with ESCC should be established.

In conclusion, preoperative GNRI can be used to sort patients into groups at high- or low-risk for shorter CSS after undergoing curative resection for ESCC, especially those with pTNM stage I and II ESCC; the interpretation should be done with care due to the differences in clinical background. Therefore, multicenter prospective validation of our findings is considered necessary to confirm the usefulness of GNRI as clinical therapeutic stratification marker for patients requiring more aggressive multimodality treatment or stringing surveillance.

## MATERIALS AND METHODS

### Patients

We retrospectively analyzed the prognostic significance of the GNRI in a total of 191 patients with ESCC who underwent curative esophagectomies, between January 2006 and October 2017, in our institute. All patients underwent thoracoscopic subtotal esophagectomies with three-field lymph node dissections, with elevation of the gastric conduit to the neck via the posterior mediastinal approach or the retrosternal approach with anastomosis of the cervical esophagus and the gastric conduit. Postoperative adjuvant and post-recurrence chemotherapy were administered according to the guidelines edited by the Japan Esophageal Society [29, 30]. According to the guidelines, preoperative chemotherapy is recommended as grade B. However, the patients who did not wish to undergo preoperative chemotherapy were preceded by surgical therapy because the current preoperative chemotherapy with 5FU and CDDP did not provide a prognostic benefit in Stage III patients. The inclusion criteria were specified as follows: 1) histologically confirmed ESCC; 2) no preoperative anticancer treatment; 3) no history of cancer or co-existence of another synchronous cancer; and 4) complete removal of ESCC without any microscopic resection margin involvement. A perioperative multidisciplinary management team including surgeons, dental hygienists, pharmacists, and nutritionists was formed to provide preoperative enteral nutrition to optimize the preoperative condition as much as possible, which aimed to reduce the incidence of postoperative complications. A blood sample used for the analysis was collected from each patient within one week before the surgery.

The study was approved by the Ethical Review Board of Shimane University Faculty of Medicine (Shimane, Japan). The requirement for informed consent was waived because of the retrospective nature of this cohort study.

### Surveillance

Postoperative follow-up evaluations were performed every 3 months for the first 2 years after the surgery, and every 6 months from the 2nd to 5th year after surgery, or until the patient died. We calculated the CSS as the date from primary esophagectomy to death due to cancer-specific causes.

### The GNRI

**Figure 4:**
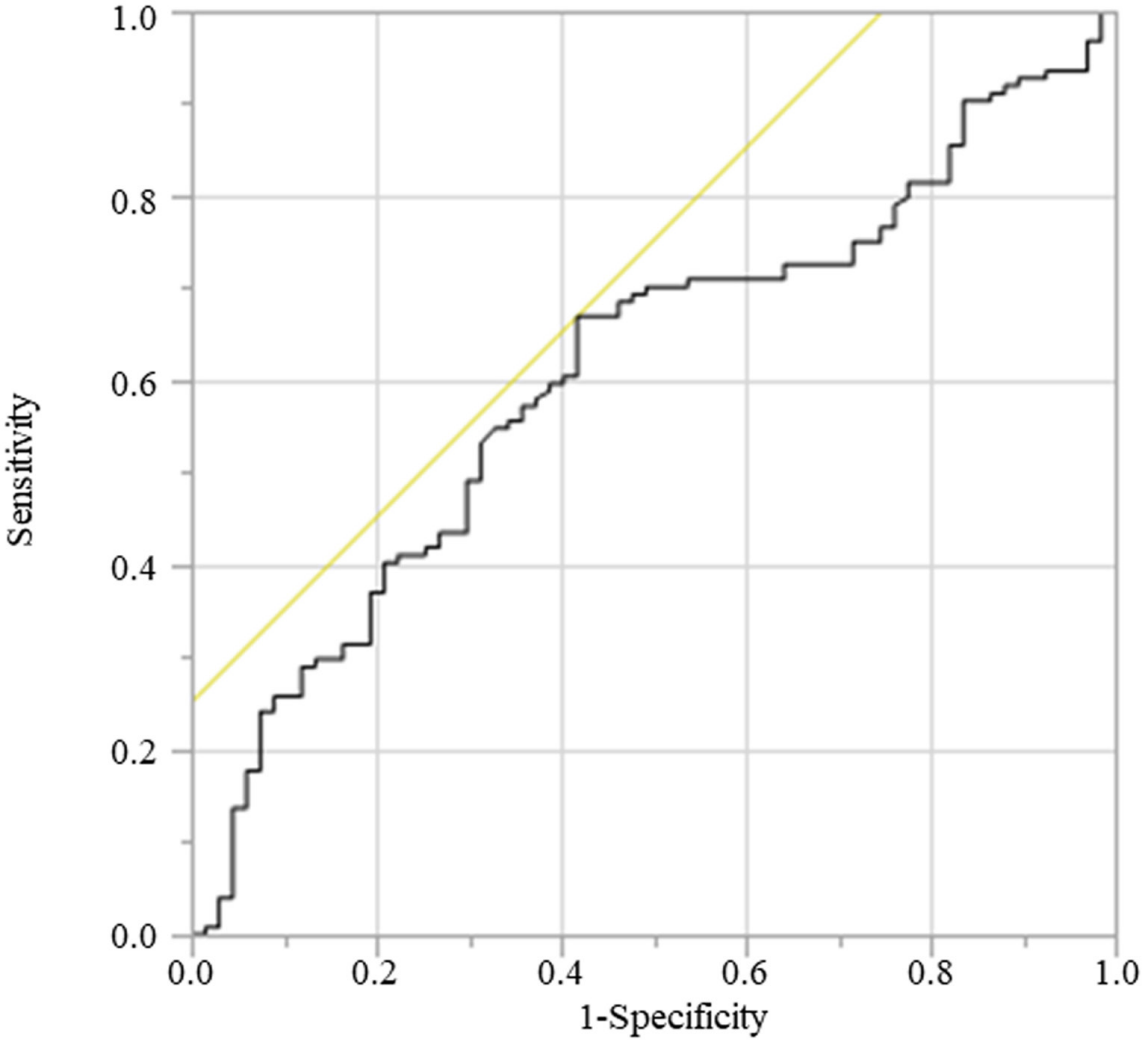
ROC for cancer death was plotted to verify the optimum cutoff of GNRI. Abbreviation: ROC, receiver operating characteristic.

The GNRI comprised two nutritional indicators: serum albumin levels and measured body weight compared with ideal body weight. The formula of GNRI was as follows: GNRI = [1.487 × serum albumin (g/L)] + [41.7 × measured/ideal body weight (BW) (kg)]. Ideal BW (kg) = 22 × square of height (m^2^) [2]. The ratio of measured BW to ideal BW was set to 1 when the measured BW of the patient exceeded the ideal BW. The ability of the GNRI to predict the CSS was assessed using a receiver operating characteristic (ROC) curve analysis, wherein the most discriminative cutoff value of GNRI was set at 97.1 with an area under curve (AUC) value of 0.608 (sensitivity, 66.9%; specificity, 58.2%) in this study (Figure 4). Patients were classified into two groups based on the cutoff value of GNRI as follows: the normal GNRI (GNRI ≥ 97.1) and low GNRI (GNRI < 97.1) groups.

### Statistical analysis

The differences between the study groups were evaluated using the Chi-square test or Student’s *t*-test for categorical variables. The Kruskal–Wallis analysis was performed for non-normally distributed continuous variables among the three groups. The CSS was calculated using the Kaplan–Meier method, and the difference between the survival curves was assessed using the log-rank test.

Variables with *p* < 0.05 in the univariate analysis were subjected to multivariate logistic regression analysis using Cox proportional hazards model. All statistical analyses were performed using the JMP software (version 14 for Windows; SAS Institute), and statistical significance was set to *p* < 0.05.

## SUPPLEMENTARY MATERIALS



## Abbreviations

AUC: area under curve
BMI: Body Mass Index
CI: Confidence Interval
CRP: C-reactive protein
CSS: Cancer-specific survival
ESCC: Esophageal squamous cell carcinoma
GNRI: geriatric nutritional risk index
GPS: Glasgow prognostic score
HR: Hazard Ratio
OS: overall survival
pTNM: pathological tumor-node-metastasis
ROC: receiver operating characteristic
SCC: squamous cell carcinoma
WBC: white blood cell
