# Supplementary material for: Geriatric nutritional risk index as a prognostic marker of pTNM-stage I and II esophageal squamous cell carcinoma after curative resection
Source: Oncotarget. 2020 Jul 21;11(29):2834–46. doi: 10.18632/oncotarget.27670 (PMC7381097; doi:10.18632/oncotarget.27670)
Supplement: Supplementary file 1 [file oncotarget-11-2834-s001.pdf]

Geriatric nutritional risk index as a prognostic marker of pTNM-stage I and II esophageal squamous cell carcinoma after curative resection

SUPPLEMENTARY MATERIALS

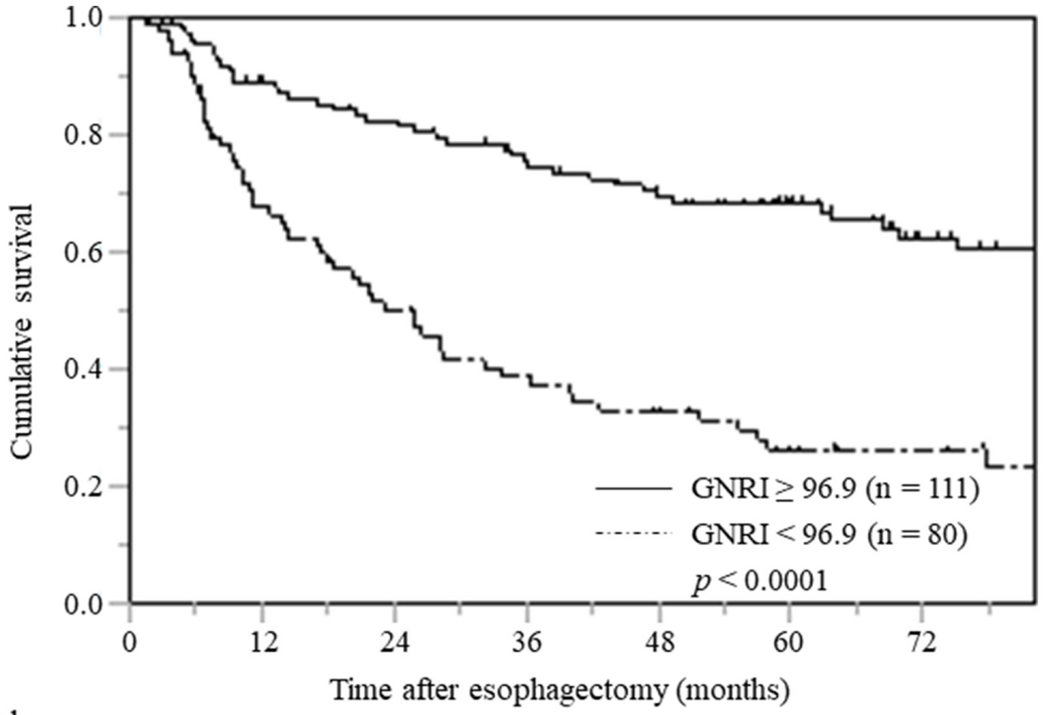

No. at risk							
GNRI ≥ 96.9	111	96	87	76	67	53	38
GNRI < 96.9	80	52	37	28	23	15	12

Supplementary Figure 1: Overall survival curves in all patients with ESCC stratified by preoperative GNRI. ROC analysis for overall death was plotted to verify the optimum cutoff of GNRI. Cutoff value of GNRI was set at 96.9 using ROC analysis for overall death (AUC value: 0.65447, sensitivity, 73.4%; specificity, 57.7%). Abbreviations: AUC, area under curve; GNRI, geriatric nutritional risk index; ROC, receiver operating characteristic.

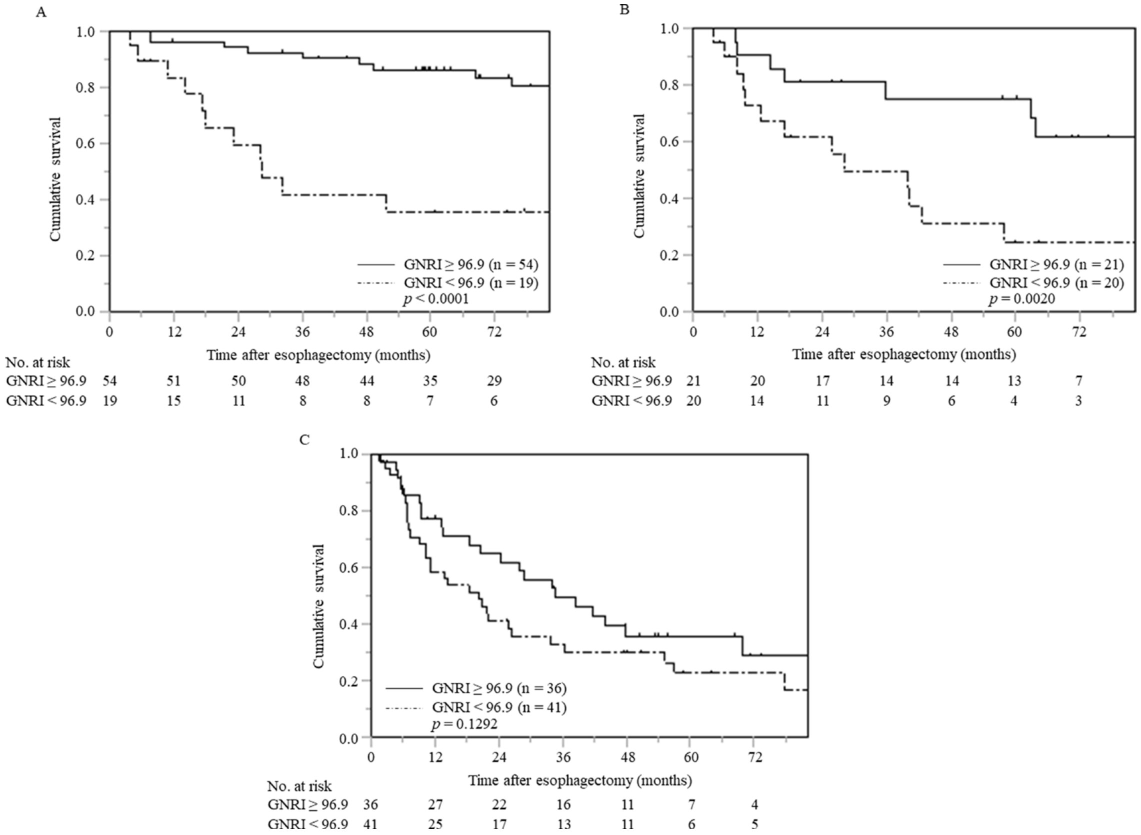

Supplementary Figure 2: Overall survival curves in patients with ESCC in each pTNM stage stratified by preoperative GNRI. (**A**) pTNM stage I (*n* = 73), (**B**) pTNM stage II (*n* = 41), (**C**) pTNM stage III (*n* = 77). ESCC, esophageal squamous cell carcinoma; pTNM, pathological tumor-node-metastasis.
